# Case Report: Godoy & Godoy method of cervical lymphatic therapy – indirect evaluation of the effect of the duration of stimulation on ocular edema

**DOI:** 10.12688/f1000research.75948.2

**Published:** 2022-04-28

**Authors:** Jose Maria Pereira de Godoy, Henrique Jose Pereira de Godoy, Ana Carolina Pereira de Godoy, Maria de Fatima Guerreiro Godoy

**Affiliations:** 1 Cardiology and Cardiovascular Surgery, Faculdade de Medicina de Sao Jose do Rio Preto, Sao Jose do Rio Preto, São Paulo, 15020010, Brazil; 2 General Surgery, Faculdade de Medicina de Sao Jose do Rio Preto, Sao Jose do Rio Preto, São Paulo, 15020010, Brazil; 3 Cardiology, Faculdade de Medicina de Sao Jose do Rio Preto, Sao Jose do Rio Preto, São Paulo, 15020010, Brazil; 4 Rehabilitation, Clínica Godoy, Sao Jose do Rio Preto, São Paulo, 15020010, Brazil

**Keywords:** Ophthalmology, Godoy & Godoy method, lymphatic therapy, glaucoma

## Abstract

The aim of the present study is to report the indirect evaluation of cervical stimulation considering the effect of the duration of the stimulus on the control of intraocular pressure in a patient with bilateral glaucoma with important ocular edema. ****A 47-year-old woman reported the onset of pain and bilateral tearing in the eyes at 35 years of age and was diagnosed with glaucoma. The patient began clinical treatment, but intraocular pressure remained 35 to 40 mmHg even with the use of four eye medications in the form of drops. The patient reported that her vision was always blurred despite the use of the eyedrops. The patient was submitted to the Godoy & Godoy method of cervical lymphatic therapy to reduce the edema. The ophthalmologist measured her intraocular pressure every two and three days. We found that the pressure was maintained below 20 mmHg when lymphatic therapy was performed every two days, but intraocular pressure increased and the vision became blurred when therapy was performed every three days. The Godoy & Godoy method of cervical lymphatic therapy constitutes a novel lymphatic system stimulation strategy that maintains its effect on intraocular pressure for approximately 48 hours, as demonstrated through an indirect evaluation.

## Introduction

The Godoy & Godoy method of cervical lymphatic therapy is a novel lymphatic stimulation concept developed in recent years based on the adaptation of the manual lymphatic drainage technique using linear movements for the treatment of facial lymphedema. ^1^^–^^3^ The method emerged from the development of a novel therapeutic option that did not involve manual drainage in the region of the carotid body to avoid the complications of its stimulation. The strategy was to drain only below the midline of the neck. However, during the treatment of a patient with cervical clearance and an ulcerated lesion below the midline with intense fibrosis, the option was to perform small sliding movements in this region. This was initially an attempt to perform linear drainage with short elongation of the skin. The following day, the patient reported improvements in both pain and neck mobility and had slept better. This information led to the decision to perform the technique 15 to 20 minutes per day. The improvement was continuous over the subsequent days, with the clinical reversal of the fibrosis, which led to an improvement in the quality of life of this patient. ^2^

Another patient with cervical clearance who was unable to close their mouth and required the use of a nasogastric tube was treated daily. After three months, the child began to eat and a dental prosthesis was adapted, leading to an improvement in quality of life. Another patient with facial edema who was unable to open the eyes and the tongue did not fit in the mouth was submitted to three days of treatment; the child was evaluated on the fifth day, at which time the patient was able to open and close the eyes and the tongue had reduced in size. ^3^

Based on these observations, the results of this method as monotherapy for lower limb lymphedema were evaluated. A two-year evaluation of this cervical method as monotherapy revealed improvement in all patients. ^4^ The assessment of this method as monotherapy for upper limb lymphedema was then performed ^5^ and a report of the results after ten years of follow-up was published. ^6^ Several studies combining this method with other forms of treatment for lymphedema have been conducted over the years. ^7^^,^^8^

A study evaluating the effects of complex decongestive physical therapy (CDP) in home programs on external lymphedema, using three-dimensional (3D) surface scanning and volume assessment in head and neck lymphedema, detects that there is a significant reduction in edema. ^9^ Study evaluating the treatment of cancer-related head and neck lymphedema using pneumatic compression concluded which is safe, easy to use and well tolerated, demonstrating reduction of edema after a single treatment. ^10^ Therefore, new therapeutic options in the treatment of lymphedema may contribute to alleviate suffering and improve quality of life.

The aim of the present study is to report the indirect evaluation of cervical stimulation considering the effect of the duration of the stimulus on the control of intraocular pressure in a patient with bilateral glaucoma with important ocular edema.

## Case report

**Figure 1.**
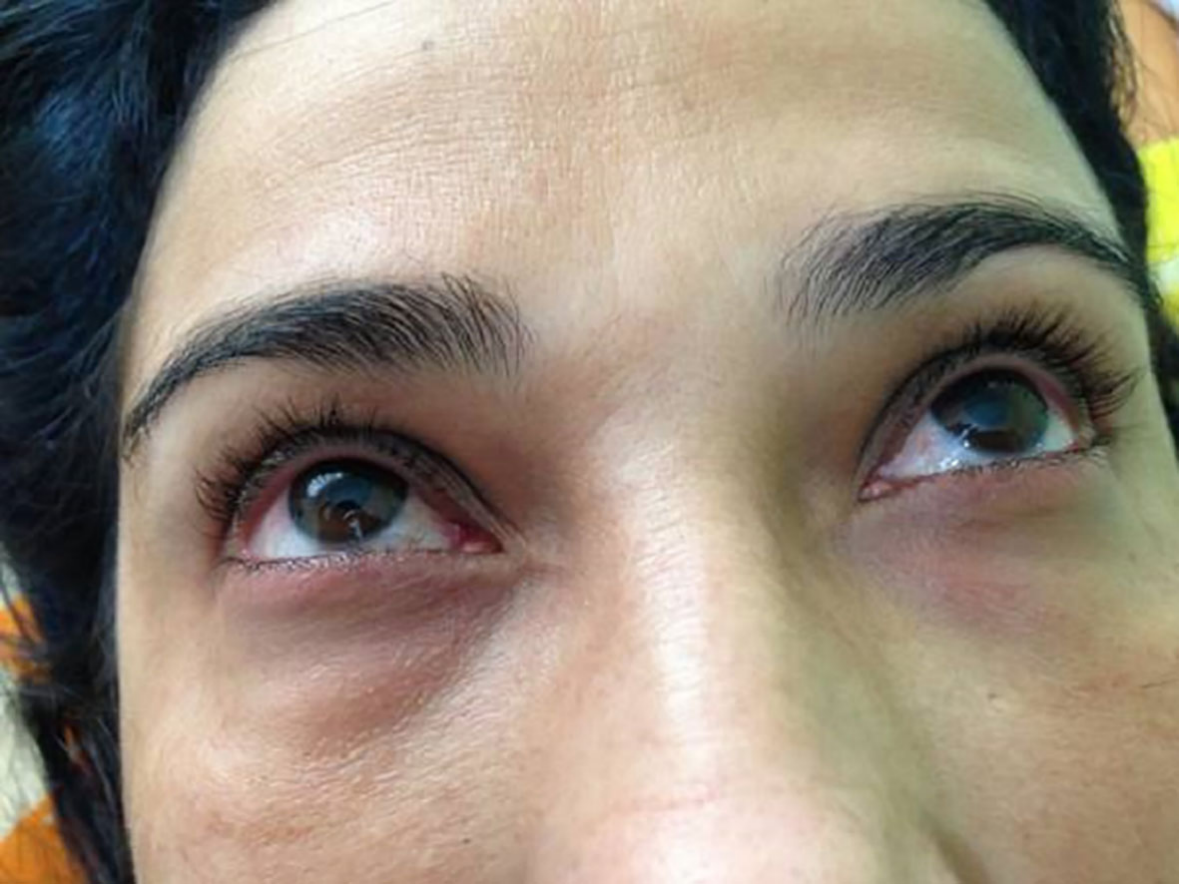
Hyperemia with periorbital edema.

**Figure 2.**
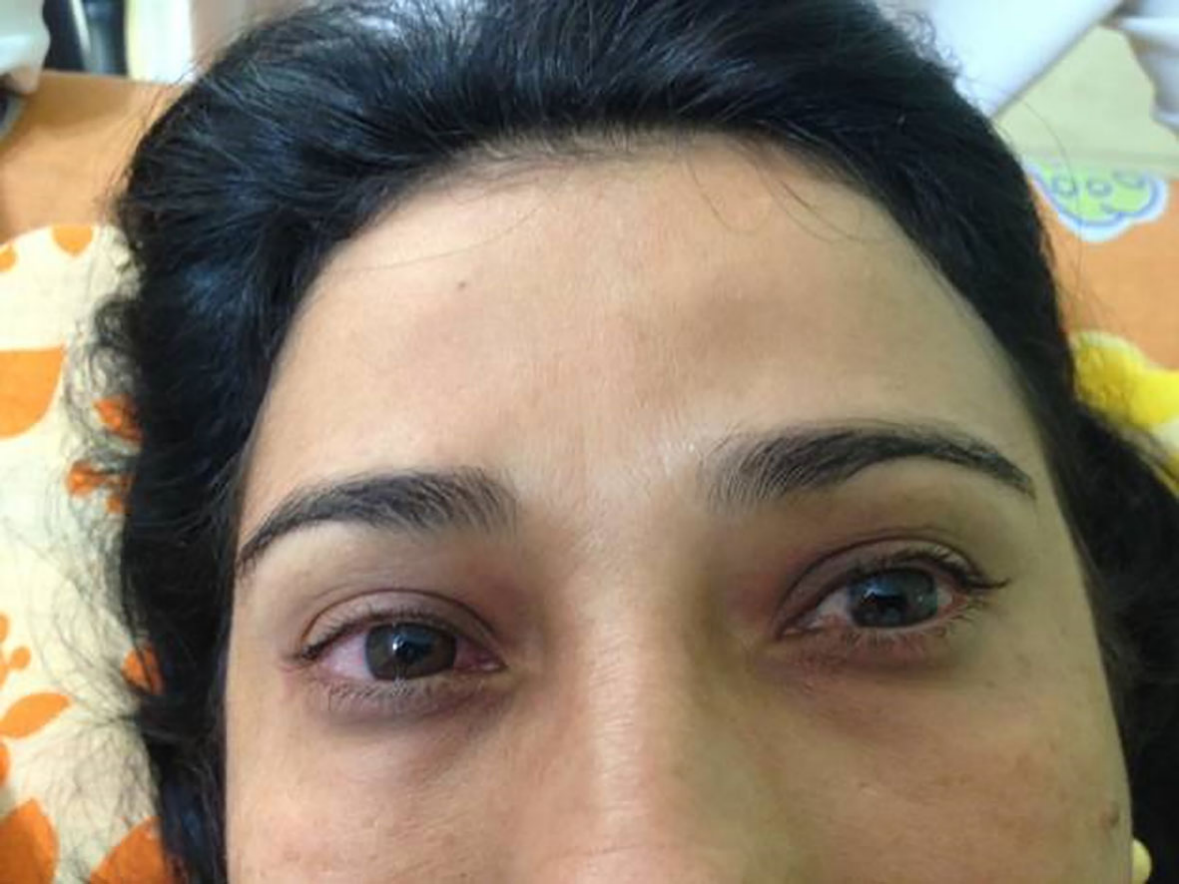
After cervical lymphatic therapy.

**Figure 3.**
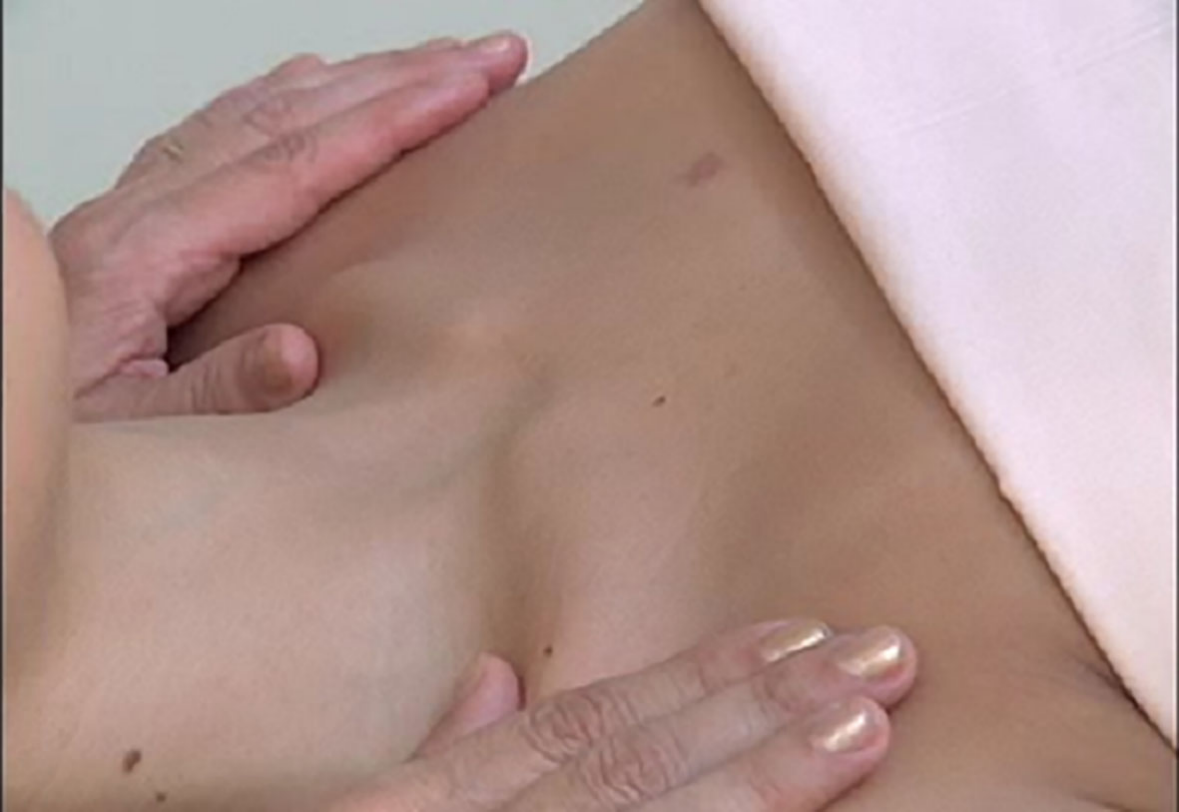
Cervical lymphatic therapy technique.

A 47-year-old woman (white, *photographer)* reported in January 2009, the onset of pain and bilateral tearing in the eyes at 35 years of age and was diagnosed with glaucoma. The patient began clinical treatment, but intraocular pressure remained 35 to 40 mmHg even with the use of four eye medications in the form of drops. She sought 13 ophthalmologists to undergo surgery for glaucoma, but none was willing to perform surgery due to the excessive edema. At 36 years of age, the patient sought a clinic for the treatment of lower limb varicose veins. CEAP C2 and hyperemia with periorbital edema were findings that drew the attention of the health team. The patient reported that her vision was always blurred despite the use of the eye drops ( Figure 1). The patient was submitted to the Godoy & Godoy method of cervical lymphatic therapy to reduce the edema ( Figure 2), which resulted in an improvement in the first session. This therapy consists of gentle elongating movements of approximately 0.5 cm on the skin surface, supraclavicular neck, at a rate of 30 movements per minute, for 20 minutes per day ( Figure 3).

The patient began to see better and no longer had blurred vision beginning with the first session. In an initial phase, cervical lymphatic therapy was performed daily and subsequently every other day. The patient’s vision was no longer blurred. Intraocular pressure was reduced to less than 20 mmHg and the ophthalmologist reduced the prescription to two medications. Periorbital edema and hyperemia were normalized. During one weekend, the patient’s vision became blurred again. The patient had spent three days without undergoing therapy. We asked the ophthalmologist to measure her intraocular pressure every two and three days. We found that the pressure was maintained below 20 mmHg when lymphatic therapy was performed every two days, but intraocular pressure increased and the vision became blurred when therapy was performed every three days.

The patient was followed up at the clinic for two years, when she was able to find an ophthalmologist to perform glaucoma surgery. Ocular pressure reduced to 7 to 8 mmHg in both eyes and remains at this level. However, even after surgery, in a few days, the vision became blurred again, usually associated with more intense physical activity, and she underwent cervical lymphatic therapy and got better again.

It has been nine years since the patient was submitted to surgery. She initially needed to perform cervical lymphatic therapy more often, but currently undergoes this therapy sporadically. Cervical lymphatic therapy maintained intraocular pressure controlled for 48 hours, but surgery brought a more lasting benefit.

The result of the vision improvement was a clinical finding well defined by the patient and the duration of the therapeutic response as well. The measurement of eye pressure was the alternative to document the association of improved vision with reduced intraocular pressure.

This study received approval from the institutional review board of the São Jose do Rio Preto School of Medicine (reference number 4.962.509), and the patient signed a consent form.

## Discussion

The present study is an indirect way of evaluating the Godoy & Godoy method of cervical stimulation, which is currently denominated the Godoy & Godoy method of cervical lymphatic therapy. The improvement in vision with the first session drew the attention of the researchers, who then performed therapy on the patient 15 to 20 minutes per day, leading to the disappearance of the blurred vision. The initial question was how to quantify these results. However, the ophthalmologist noted an improvement in intraocular pressure from approximately 40 mmHg to less than 20 mmHg, with the reduction from four eye medications to two.

Daily therapy led to the maintenance of non-blurred vision, but the vision blurred when the patient performed physical effort, which led to an increase in intraocular pressure. Another important observation was the fact that the patient’s vision became blurred when she spent three days without cervical lymphatic therapy and improved again when returning to therapy. Thus, the decision was made to standardize the ocular evaluation every two and three days, which revealed that normal vision was maintained for two days and became blurred on the third day due to the increase in intraocular pressure, suggesting that cervical lymphatic therapy maintains the results for approximately 48 hours. Therefore, this is a novel form of stimulating the lymphatic system that maintains its effects for 48 hours. One of the hypotheses is neurological stimulus, which remains activated for various hours.

This experiment was conducted several times over a two-year period until the patient was able to undergo glaucoma surgery. However, her vision frequently becomes blurred and improves with cervical lymphatic therapy, which was initially required more often and is currently only needed sporadically. The Godoy & Godoy method of cervical lymphatic therapy constitutes a novel lymphatic system stimulation strategy that maintains its effect on intraocular pressure for approximately 48 hours, as demonstrated through an indirect evaluation, but further studies are needed to confirm more lasting benefit and for similar cases.

## Data availability

All data underlying the results are available as part of the article and no additional source data are required.

## Consent

Written informed consent for publication of their clinical details and clinical images was obtained from the patient.
