# Case Report: Godoy & Godoy method of cervical lymphatic therapy – indirect evaluation of the effect of the duration of stimulation on ocular edema

**DOI:** 10.12688/f1000research.75948.3

**Published:** 2022-05-30

**Authors:** Jose Maria Pereira de Godoy, Henrique Jose Pereira de Godoy, Ana Carolina Pereira de Godoy, Maria de Fatima Guerreiro Godoy

**Affiliations:** 1 Cardiology and Cardiovascular Surgery, Faculdade de Medicina de Sao Jose do Rio Preto, Sao Jose do Rio Preto, São Paulo, 15020010, Brazil; 2 General Surgery, Faculdade de Medicina de Sao Jose do Rio Preto, Sao Jose do Rio Preto, São Paulo, 15020010, Brazil; 3 Cardiology, Faculdade de Medicina de Sao Jose do Rio Preto, Sao Jose do Rio Preto, São Paulo, 15020010, Brazil; 4 Rehabilitation, Clínica Godoy, Sao Jose do Rio Preto, São Paulo, 15020010, Brazil

**Keywords:** Ophthalmology, Godoy & Godoy method, lymphatic therapy, glaucoma

## Abstract

The aim of the present study is to report the indirect evaluation of cervical stimulation considering the effect of the duration of the stimulus on the control of intraocular pressure in a patient with bilateral glaucoma with important ocular edema. ****A 47-year-old woman reported the onset of pain and bilateral tearing in the eyes at 35 years of age and was diagnosed with glaucoma. The patient began clinical treatment, but intraocular pressure remained 35 to 40 mmHg even with the use of four eye medications in the form of drops. The patient reported that her vision was always blurred despite the use of the eyedrops. The patient was submitted to the Godoy & Godoy method of cervical lymphatic therapy to reduce the edema. The ophthalmologist measured her intraocular pressure every two and three days. We found that the pressure was maintained below 20 mmHg when lymphatic therapy was performed every two days, but intraocular pressure increased and the vision became blurred when therapy was performed every three days. The Godoy & Godoy method of cervical lymphatic therapy constitutes a novel lymphatic system stimulation strategy that maintains its effect on intraocular pressure for approximately 48 hours, as demonstrated through an indirect evaluation.

## Introduction

The Godoy & Godoy method of cervical lymphatic therapy is a novel lymphatic stimulation concept developed in recent years based on the adaptation of the manual lymphatic drainage technique using linear movements for the treatment of facial lymphedema. ^1^ ^–^ ^3^ The method emerged from the development of a novel therapeutic option that did not involve manual drainage in the region of the carotid body to avoid the complications of its stimulation. The strategy was to drain only below the midline of the neck by means of small sliding movements in this region and linear drainage with short elongation of the skin. A few specific cases of facial, month and eye edema were previously treated with this method achieving positive outcomes. ^3^ Furthermore, based on these observations, of this method as monotherapy for lower and upper limb lymphedema was successfully applied as well. ^4^ ^,^ ^5^ Interestingly a report of long term (10 years) results of this protocol in congenital lymphedema was recently published. ^6^ Several studies combining this method with other forms of treatment for lymphedema have been conducted over the years. ^7^ ^,^ ^8^ A study evaluating the effects of complex decongestive physical therapy (CDP) in home programs on external lymphedema, using three-dimensional (3D) surface scanning and volume assessment in head and neck lymphedema, detects that there is a significant reduction in edema. ^9^ Study evaluating the treatment of cancer-related head and neck lymphedema using pneumatic compression concluded which is safe, easy to use and well tolerated, demonstrating reduction of edema after a single treatment. ^10^ Therefore, new therapeutic options in the treatment of lymphedema may contribute to alleviate suffering and improve quality of life. The aim of the present study is to report the indirect evaluation of cervical stimulation considering the effect of the duration of the stimulus on the control of intraocular pressure in a patient with bilateral glaucoma with concomitant relevant ocular edema.

## Case report

A 47-year-old woman white, reported that in January 2009, the onset of pain and bilateral tearing in the eyes at 35 years of age and was diagnosed with glaucoma. The patient began conventional clinical treatment, with little benefit and intraocular pressure remained in the level of 35 to 40 mmHg even with the use of four eye medications in the form of drops. She sought 13 ophthalmologists to assess the possibility to undergo surgery for her glaucoma, but the excessive edema was considered a contraindication. When she was 36 years old, the patient presented to our clinic with hyperemia and periorbital edema. The patient reported a blurred despite the use of the eye drops ( Figure 1A). Hence the patient was submitted to the Godoy & Godoy method of cervical lymphatic therapy to reduce the edema ( Figure 1B), which resulted in an improvement since the first session. The therapy consists of gentle elongating movements of approximately 0.5 cm on the skin surface, supraclavicular neck, at a rate of 30 movements per minute, 20 minutes per day, Figure 1C.

**Figure 1A.**
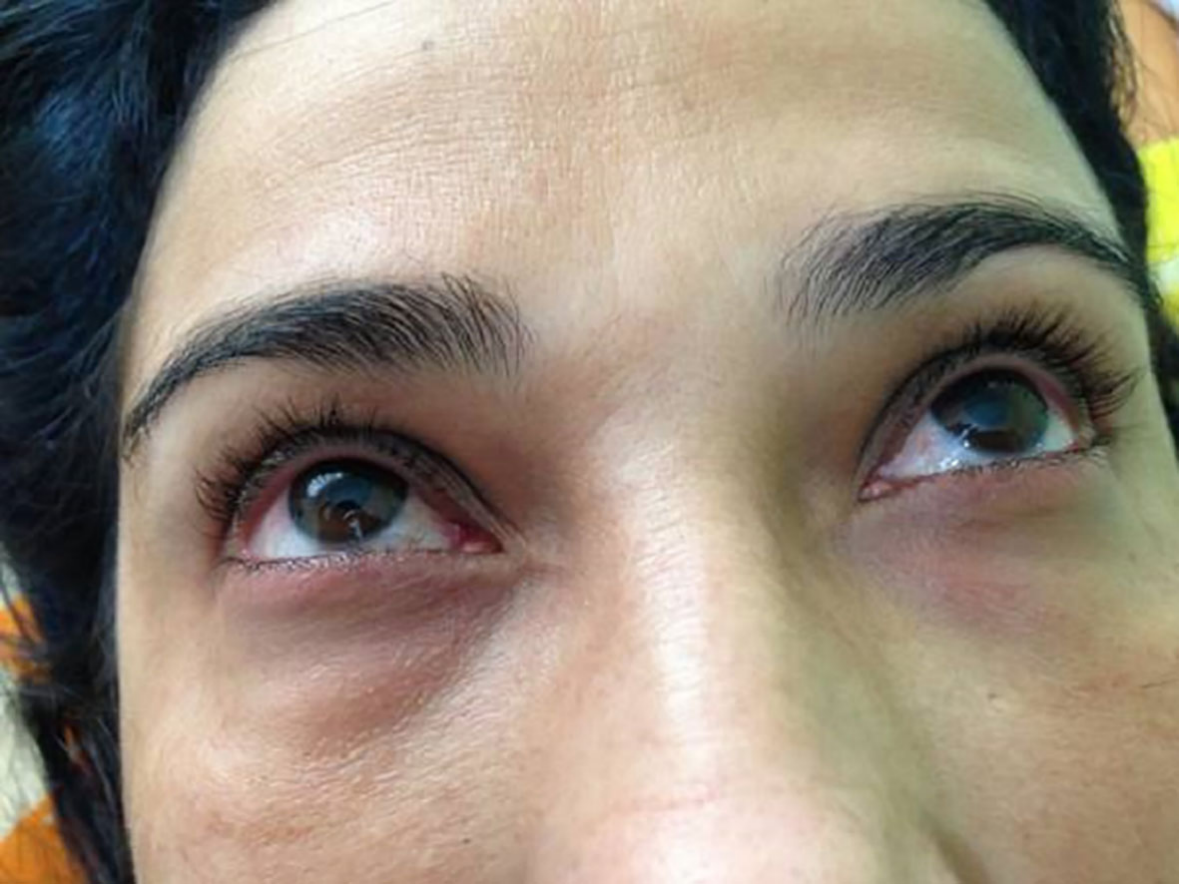
Hyperemia with periorbital edema.

**Figure 1B.**
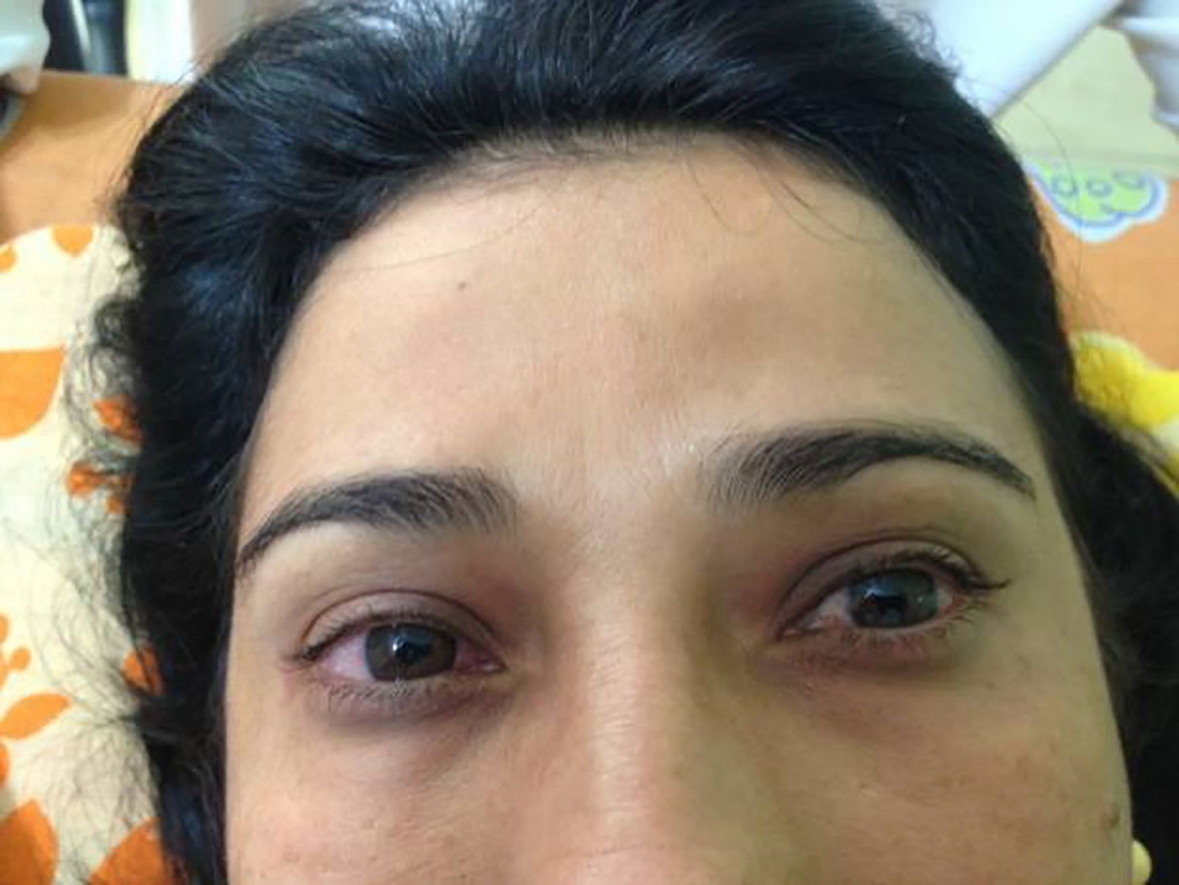
After cervical lymphatic therapy.

**Figure 1C.**
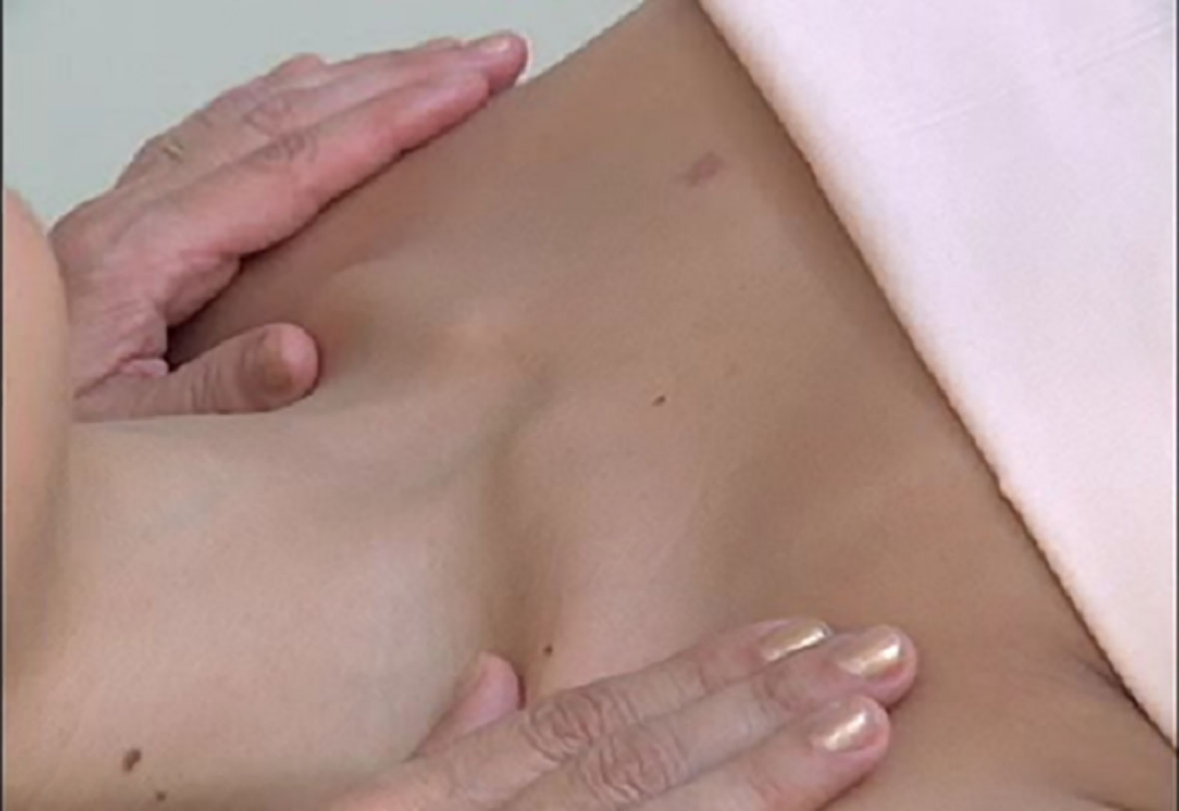
Cervical lymphatic therapy technique.

The patient improved her sight and she referred no more blurred vision. In an initial phase, cervical lymphatic therapy was performed daily and subsequently every other day. Together with the clinical improvement the ophthalmologist recorded a decrease of intraocular pressure to the value of less than 20 mmHg and the pharmacological treatment was reduced to two medications. Periorbital edema and hyperemia were normalized.

When interrupting the treatment, the patient experienced a clinical worsening again. Once re-started the treatment protocol the ophthalmologist was asked to measure her intraocular pressure, after each treatment. I was found the pressure was maintained below 20 mmHg when lymphatic therapy was performed every two days, but intraocular pressure increased and the vision became blurred when therapy was performed every three days.

The patient was followed up at the clinic for two years, until she was submitted to surgery for her glaucoma. Ocular pressure consequently reduced to 7 to 8 mmHg in both eyes and remains at this level. However, even after surgery, the vision deteriorated, again afterwards which led us to cervical lymphatic therapy achieving a clinical improvement.

It has been nine years since the patient was submitted to surgery. She initially needed to perform cervical lymphatic therapy more often, but currently she undergoes this therapy less frequently. In conclusion the application of cervical lymphatic therapy in a glaucoma patient proved effective to maintain intraocular pressure under control for 48 hours; similarly surgery resulted in longer lasting benefit, thought complementary manual therapy can be equally indicated to improve the surgical outcomes furthermore. This study received approval from the institutional review board of the São Jose do Rio Preto School of Medicine (reference number 4.962.509), and the patient signed a consent form.

The patient began to see better and no longer had blurred vision beginning with the first session. In an initial phase, cervical lymphatic therapy was performed daily and subsequently every other day. The patient’s vision was no longer blurred. Intraocular pressure was reduced to less than 20 mmHg and the ophthalmologist reduced the prescription to two medications. Periorbital edema and hyperemia were normalized. During one weekend, the patient’s vision became blurred again. The patient had spent three days without undergoing therapy. We asked the ophthalmologist to measure her intraocular pressure every two and three days. We found that the pressure was maintained below 20 mmHg when lymphatic therapy was performed every two days, but intraocular pressure increased and the vision became blurred when therapy was performed every three days.

The patient was followed up at the clinic for two years, when she was able to find an ophthalmologist to perform glaucoma surgery. Ocular pressure reduced to 7 to 8 mmHg in both eyes and remains at this level. However, even after surgery, in a few days, the vision became blurred again, usually associated with more intense physical activity, and she underwent cervical lymphatic therapy and got better again.

It has been nine years since the patient was submitted to surgery. She initially needed to perform cervical lymphatic therapy more often, but currently undergoes this therapy sporadically. Cervical lymphatic therapy maintained intraocular pressure controlled for 48 hours, but surgery brought a more lasting benefit.

The result of the vision improvement was a clinical finding well defined by the patient and the duration of the therapeutic response as well. The measurement of eye pressure was the alternative to document the association of improved vision with reduced intraocular pressure.

This study received approval from the institutional review board of the São Jose do Rio Preto School of Medicine (reference number 4.962.509), and the patient signed a consent form.

## Discussion

The present study is an indirect way of evaluating the Godoy & Godoy method of manual cervical stimulation, which is currently denominated the Godoy & Godoy method of cervical lymphatic therapy. This specific treatment proved effective to improve eye vision in our glaucoma patient. In order to quantify also the possible results of this method on the intraocular pressure, specific measurements were performed and showed a decrease from approximately 40 mmHg to less than 20 mmHg. The patient could also discontinue two of her medication and daily therapy led to the maintenance of non-blurred vision, except when the patient performed physical effort, which led to an increase in intraocular pressure. It was also noted that the patient’s vision typically deteriorated when she spent three days without cervical lymphatic therapy and improved again when receiving the therapy again. Hence the decision was made to standardize the ocular evaluation every two and three days. The ophthalmologist’s examinations revealed that cervical lymphatic therapy maintains the results for approximately 48 hours. Therefore, this novel form of stimulating the lymphatic system may maintain its effects for 48 hours. Of the hypotheses is that the induced longer-lasting neurological stimulus may contribute to this effect.

Overall, the Godoy & Godoy method of cervical manual lymphatic therapy constitutes a novel strategy to induce lymphatic system stimulation which maintains its effect on intraocular pressure for approximately 48 hours, as demonstrated through an indirect evaluation. Further studies are needed to confirm more lasting benefit and for similar cases.

## Data availability

All data underlying the results are available as part of the article and no additional source data are required.

## Consent

Written informed consent for publication of their clinical details and clinical images was obtained from the patient.
