# Supplementary material for: Case Report: Godoy & Godoy method of cervical lymphatic therapy – indirect evaluation of the effect of the duration of stimulation on ocular edema
Source: F1000Res. 2022 May 30;11:112. Originally published 2022 Jan 28. [Version 3] doi: 10.12688/f1000research.75948.3 (PMC9174690; doi:10.12688/f1000research.75948.3)
Supplement: Peer review history

# Peer review: Reviewer response for version 3

**DOI:** 10.5256/f1000research.134016.r139415

**Published:** 2022-06-07

**Authors:** Attilio Cavezzi

**Affiliations:** 1 Eurocenter Venalinfa, San Benedetto del Tronto, Italy

The authors revised the text of the manuscript, which brings an improvement in the understanding and readability of the text. Overall the manuscript is approved for indexing, provided an English text revision is finalised.

 Moreover, a few sentences are repeated throughout the manuscript and should be removed, giving the text a continuous flow, etc. For example, these two sentences and the related phrasing are to be checked and the duplication (from the old text) removed:

 " *The patient was followed up at the clinic for two years, when she was able to find an ophthalmologist to perform glaucoma surgery. Ocular pressure reduced to 7 to 8 mmHg in both eyes and remains at this level. However, even after surgery, in a few days, the vision became blurred again, usually associated with more intense physical activity, and she underwent cervical lymphatic therapy and got better again. It has been nine years since the patient was submitted to surgery. She initially needed to perform cervical lymphatic therapy more often, but currently undergoes this therapy sporadically. Cervical lymphatic therapy maintained intraocular pressure controlled for 48 hours, but surgery brought a more lasting benefit. The result of the vision improvement was a clinical finding well defined by the patient and the duration of the therapeutic response as well. The measurement of eye pressure was the alternative to document the association of improved vision with reduced intraocular pressure. This study received approval from the institutional review board of the São Jose do Rio Preto School of Medicine (reference number 4.962.509), and the patient signed a consent form*."

Are enough details provided of any physical examination and diagnostic tests, treatment given and outcomes?

Yes

Is the case presented with sufficient detail to be useful for other practitioners?

Yes

Is sufficient discussion included of the importance of the findings and their relevance to future understanding of disease processes, diagnosis or treatment?

Yes

Is the background of the case’s history and progression described in sufficient detail?

Partly

Reviewer Expertise:

Vascualr Surgery, vascular medicine, longevity medicine, psychoneuroendocrineimmunology,

I confirm that I have read this submission and believe that I have an appropriate level of expertise to confirm that it is of an acceptable scientific standard.

# Peer review: Reviewer response for version 1

**DOI:** 10.5256/f1000research.79894.r127076

**Published:** 2022-03-29

**Authors:** Attilio Cavezzi

**Affiliations:** 1 Eurocenter Venalinfa, San Benedetto del Tronto, Italy

The authors report their experience with an original manual method to improve lymphatic drainage in a selective indication. This case-report correctly indicates the possibilities and limitations of manual lymphatic drainage in these specific cases and the authors have presented their own method, with interesting outcomes. In order to facilitate the review of the text, a detailed proposal of text revision is provided below  Lastly, some more references regarding head and neck manual lymphatic therapy could be added in the introduction and/on discussion section. For this purpose, a link with a few references is provided here: https://pubmed.ncbi.nlm.nih.gov/?term=manual+lymph+drainage+neck+face&sort=date

 Here is my proposed revision: Godoy Review

Are enough details provided of any physical examination and diagnostic tests, treatment given and outcomes?

Yes

Is the case presented with sufficient detail to be useful for other practitioners?

Yes

Is sufficient discussion included of the importance of the findings and their relevance to future understanding of disease processes, diagnosis or treatment?

Yes

Is the background of the case’s history and progression described in sufficient detail?

Partly

Reviewer Expertise:

Vascualr Surgery, vascular medicine, longevity medicine, psychoneuroendocrineimmunology,

I confirm that I have read this submission and believe that I have an appropriate level of expertise to confirm that it is of an acceptable scientific standard, however I have significant reservations, as outlined above.

# Response

**Published:** 2022-03-30

**Authors:** Jose Maria Pereira de Godoy

**Affiliations:** Faculdade de Medicina de Sao Jose do Rio Preto, Brazil

The authors report their experience with an original manual method to improve lymphatic drainage in a selective indication. This case-report correctly indicates the possibilities and limitations of manual lymphatic drainage in these specific cases and the authors have presented their own method, with interesting outcomes. In order to facilitate the review of the text, a detailed proposal of text revision is provided below  Lastly, some more references regarding head and neck manual lymphatic therapy could be added in the introduction and/on discussion section. For this purpose, a link with a few references is provided here:  https://pubmed.ncbi.nlm.nih.gov/?term=manual+lymph+drainage+neck+face&sort=date

***Reply: The authors added references in the introduction.***

 Here is my proposed revision: Godoy Review

 Is the background of the case’s history and progression described in sufficient detail?

 Partly

***Reply: The authors added.***

 Are enough details provided of any physical examination and diagnostic tests, treatment given and outcomes?

 Yes

***Reply: thanks.***

 Is sufficient discussion included of the importance of the findings and their relevance to future understanding of disease processes, diagnosis or treatment?

 Yes

 Is the case presented with sufficient detail to be useful for other practitioners?

 Yes

***Reply: thanks.***

# Peer review: Reviewer response for version 1

**DOI:** 10.5256/f1000research.79894.r121447

**Published:** 2022-02-08

**Authors:** Evangelos Dimakakos

**Affiliations:** 1 Oncology Unit, 3rd Department of Medicine, 'Sotiria' Hospital for Chest Diseases, National and Kapodistrian University of Athens, Athens, Greece

The paper entitled Case Report: Godoy & Godoy method of cervical lymphatic therapy – indirect evaluation of the effect of the duration of stimulation on ocular edema,  is a case report paper very well written and explains a novel therapeutic option on ocular edema. The background of the case’s history is described in sufficient detail, the method and the explanation of the treatment and its results are very clearly described. In the discussion, the authors analyzes with a clear way the importance of the treatment and the results of this new therapeutic method in a way that is very useful for other practitioners.

Are enough details provided of any physical examination and diagnostic tests, treatment given and outcomes?

Yes

Is the case presented with sufficient detail to be useful for other practitioners?

Yes

Is sufficient discussion included of the importance of the findings and their relevance to future understanding of disease processes, diagnosis or treatment?

Yes

Is the background of the case’s history and progression described in sufficient detail?

Yes

Reviewer Expertise:

Lymphedema  VTE AND cancer

I confirm that I have read this submission and believe that I have an appropriate level of expertise to confirm that it is of an acceptable scientific standard.
